# Constructing Continuous Proton-Conducting Highways within Sulfonated Poly(Arylene Ether Nitrile) Composite Membrane by Incorporating Amino-Sulfo-Bifunctionalized GO

**DOI:** 10.3390/polym10091005

**Published:** 2018-09-10

**Authors:** Tao Cheng, Xuechun Zhang, Yan Ma, Yumin Huang, Xiaobo Liu

**Affiliations:** Research Branch of Advanced Functional Materials, School of Materials and Energy, and Center for Applied Chemistry, University of Electronic Science and Technology of China, Chengdu 611731, China; chengtao94@163.com (T.C.); 18483697269@163.com (X.Z.); mayan2765@163.com (Y.M.)

**Keywords:** sulfonated poly(arylene ether nitrile), amino-sulfo-bifunctionalized GO, proton exchange membrane, proton conductivity

## Abstract

To obtain a proton exchange membrane (PEM) with high proton conductivity and low methanol permeability, a novel amino-sulfo-bifunctionalized GO (NSGO) was synthesized and explored as a filler for sulfonated poly(arylene ether nitrile) (SPEN). The result indicated that the microstructure of composite membranes was rearranged by NSGO and strong acid–base interactions were formed between fillers and the SPEN matrix, affording enhanced thermal, mechanical, and dimensional stabilities. Moreover, it was found that NSGO fillers were uniformly dispersed in the SPEN matrix, generating efficient proton-conducting paths along the SPEN/NSGO interface. Meanwhile, the sulfonic and amino groups of NSGO served as additional proton hopping sites to connect the ionic clusters in the SPEN matrix, creating interconnected and long-range ionic pathways. In such a way, proton-conducting highways with low energy barriers are constructed, which enhance the proton conductivity of the composite membranes via the Grotthuss mechanism. Furthermore, the composite membranes also effectively prevent methanol permeation, and therefore high selectivity (the ratio of proton conductivity and methanol permeability) is endowed. Compared to SPEN membrane, a 3.6-fold increase in selectivity is obtained for the optimal composite membrane. This study will provide a new strategy for the preparation of high-performance PEM.

## 1. Introduction

In recent years, direct methanol fuel cells (DMFCs) have attracted much attention for their high efficiency and low operating temperature [1]. In addition, methanol, as the fuel, shows higher energy density and higher safety of operation and transportation than other fuels, such as hydrogen. The proton exchange membrane (PEM) plays an important role as the heart of DMFCs, which separates the fuel from the oxidant and acts as the electrolyte [2].

Currently, perflurosulfonic acid polymer membranes (e.g., Nafion) are the most common commercially available PEMs, owing to their high proton conductivity and outstanding chemical stability [3]. Unfortunately, high price and methanol permeability limit their applications. Since the water molecules in the membrane can simultaneously promote proton conduction and methanol permeability, high proton conductivity is often accompanied by high methanol permeability. Therefore, many efforts have been stimulated to develop new strategies for a good trade-off effect between proton conduction and methanol permeability.

Organic–inorganic hybrids are considered to be a simple and effective approach to solve this problem [4,5]. Organic–inorganic hybrid membranes integrate the merits of both organic and inorganic components. Moreover, the performance of the hybrid membranes could be improved significantly through the synergistic effect of organic and inorganic components. Some inorganic fillers, such as organomodified silica, zeolite, titanium oxide, and graphene oxide, have been investigated to improve the performance of polymer-based composites for DMFC [6,7,8,9]. Among these candidates, graphene oxide (GO), as the representative of two-dimensional (2D) materials, has received wide attention in the field of PEMs due to its fascinating properties [10]. Its abundant oxygen functional groups and large surface area contribute to proton conduction [11]. Meanwhile, its nanosheets also possess outstanding resistance to methanol permeation [12]. However, poor interface compatibility between GO and polymer matrix may affect the performance of the composite membrane.

Based on this point, various functionalized GOs have been synthesized to attain homogeneous dispersion in a polymer matrix [10]. Usually, acid and basic groups are designed to modify GO, so as to tailor the structure and performance of the composite membrane. For example, sulfonic-functionalized GO has been widely used as filler to embed into polymer matrix [13,14]. The introduction of sulfonic groups endows the composite membrane with more proton-conducting sites to improve proton conduction. In addition, amino groups have also been designed and introduced into GO and then incorporated into the polymer matrix, in the so-called acid–base composite membrane [15,16,17]. The N atoms act as proton receptors and proton donors, resulting in the shortening of the proton transmission distance and fast proton migration with low energy barriers. More importantly, the acid–base interactions formed by *N*-containing groups and sulfonic groups can also reduce the swelling and methanol permeability of the membrane. In addition, our previous work found that the synergistic effect of codoped sulfonic-functionalized GO and amino-functionalized GO can greatly enhance the performance of the PEM [18]. However, the functionalized GO containing both sulfonic (–SO_3_H) and amino groups (–NH_2_) is still unexplored.

Usually, the functionalized GO is prepared mainly by means of physical adsorption [19] or chemical grafting [13,14]. Compared with the method of physical adsorption, chemical grafting can make the anchoring effect more stable. However, the limitation of grafting reactions restricts the type of functional groups. Therefore, developing a simple and useful method for obtaining the functionalized GO containing sulfonic and amino groups is highly required.

In this study, sulfonic and amino groups are introduced into GO by using aryl diazonium salt. Sulfonated poly(arylene ether nitrile) (SPEN) is chosen as the polymer matrix because of its excellent mechanical properties and thermal stability. In addition, the strong polar nitrile groups of SPEN could reduce swelling and enhance the adhesion property between the electrode layers for a long duration in DMFC application [14,20,21]. Then, the functionalized GO is embedded into a SPEN matrix to prepare stable acid–base composite membranes. The microstructures and physicochemical properties of the composite membranes are investigated in detail. Moreover, the proton conduction is studied systematically and a proton-conducting mechanism is proposed. Meanwhile, the effect of acid–base interactions on methanol permeability is also revealed to balance the trade-off effect of proton conduction.

## 2. Experimental

### 2.1. Materials

2,5-Diaminobenzenesulfonic acid, sodium nitrite (NaNO_2_), hydrochloric acid (HCl), sodium hydroxide (NaOH), toluene, dimethylacetamide (DMAc), sulfuric acid (H_2_SO_4_) and *N*-methylpyrrolidone (NMP) were supplied from Chengdu Kelong Chemical Reagent Company (Chengdu, China). Graphene was obtained by XFNANO Materials Tech Co. Ltd. (Nanjing, China). All the reagents were used without further treatment. The preparation of SPEN was conducted according to the previous work [18]. GO was synthesized by the improved Hummer’s method [14].

### 2.2. Preparation of Amino-Sulfo-Bifunctionalized GO (NSGO)

**Scheme 1:**
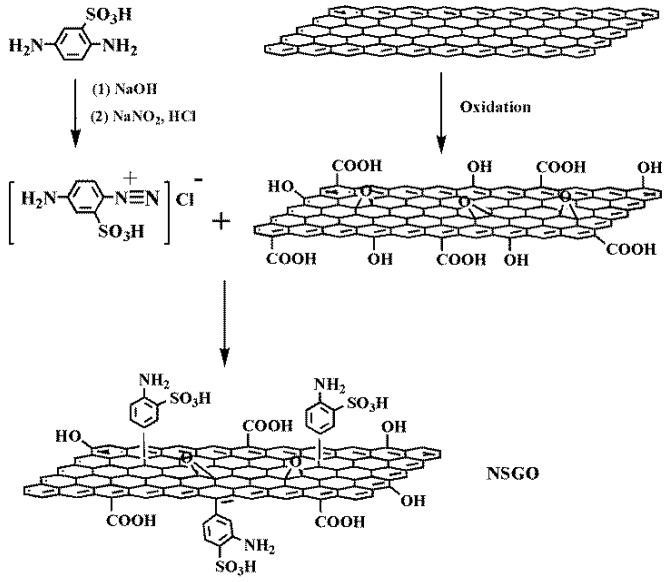
The schematic diagram of NSGO synthesis.

NSGO was synthesized from aryl diazonium salt. In a 100 mL flask, 2,5-diaminobenzenesulfonic acid was completely dissolved in 10 mL NaOH solution (8%) in a warm water bath. Then, 8 mL NaNO_2_ solution (7%), 10 mL HCl, and 10 mL water were successively added under stirring. The mixed solution was cooled to 0–5 °C and kept vigorously stirred for 15 min to form the diazonium salt. The obtained solution was injected dropwise into 40 mL GO solution (5 mg mL^−1^). After strongly stirring for 4 h in an ice water bath, the target product, NSGO, was collected by filtration, washed several times with deionized water, and dried in vacuum for 24 h at 80 °C. The schematic diagram of NSGO synthesis is shown in Scheme 1.

### 2.3. Preparation of the Membranes

**Scheme 2:**
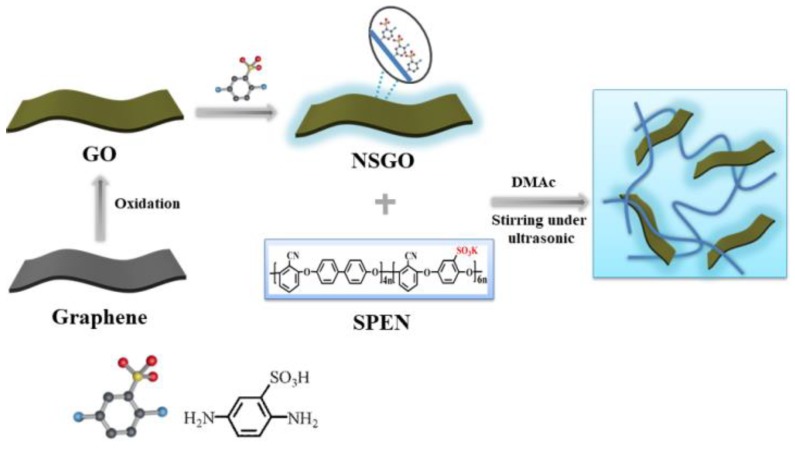
The preparation of the composite membranes.

Typically, SPEN/NSGO composite membranes were prepared by a facile solution-casting method. The various amounts of NSGO fillers (0.5, 1, 2, 3 wt %) were dispersed in 5 mL DMAc under ultrasonication for 2 h to achieve homogeneous dispersion. Meanwhile, SPEN was dissolved in 15 mL DMAc to form a homogeneous and transparent solution. Then, the NSGO solution was added into the SPEN solution and kept under ultrasonic treatment for another 2 h to obtain a uniform solution. Finally, the mixture solution was cast onto a clean and horizontal glass plate. After evaporating the solvent in a vacuum oven (80, 100, 120, 140, and 160 °C each for 2 h), the SPEN/NSGO composite membranes were obtained. The obtained membranes were immersed in 1 M H_2_SO_4_ for 24 h. Then, they were immersed in deionized water to remove the excess of acids. During this period, the deionized water was replaced every two hours until the pH value reached 6.5–7.0. The obtained membranes were designated as SPEN/NSGO-X (X represents the weight percentage of NSGO). For a comparison, a SPEN membrane was fabricated in the same way. The schematic diagram of the preparation of composite membranes is shown in Scheme 2.

### 2.4. Characterizations

Fourier transform infrared spectroscopy spectra (FTIR) were recorded using a Nicolet IS10 (Thermo Fisher, Waltham, MA, USA). The powder samples were pelleted with KBr and analyzed in transmission mode, and the samples (the size of the membrane is 1.5 cm × 1.5 cm) were analyzed in total reflection mode. Thermogravimetric analysis of the samples (the weight of the membrane is about 5–10 mg) was conducted on a TA Instruments Q50 (TA Instruments Ltd., New Castle, DE, USA) under N_2_ (Chengdu Chen Yuan Gas Co., Ltd., Chengdu, China) atmosphere (flow rate of 60 mL·min^−1^) over the temperature range of 20–600 °C at a heating rate of 20 °C/min. X-ray diffraction (XRD) was performed in a BRUCKER D8 diffractometer (Bruker, Karlsruhe, Germany) with Cu-Kα radiation to analysis the structure of GO and SNGO, and the interlayer spacing was calculated using the following equation: λ= 2d·sin θ. X-ray photoelectron spectroscopic (XPS) was conducted on an ESCA 2000 (VG Instruments, Manchester, UK) with Al-Kα radiation. The cross-sectional morphologies of all membranes were observed by using SEM (FEI INSPECT F50, Hillsboro, OR, USA). Prior to testing, the membranes were fractured in liquid nitrogen and sputtered with gold to obtain a thin and conductive surface. The mechanical properties of the samples (the size of the membrane is 1 cm × 15 cm) at fully wet state were investigated by a desktop electromechanical universal testing machine (SANS CMT6104, Shenzhen Sans Materials Testing Machine Co., Shenzhen, China) with an elongation rate of 5 mm·min^−1^ at room temperature. Atomic force microscopy (AFM) of the samples (the size of the membrane is 1 cm × 1 cm) were carried out on a Dimension Icon (Bruker Nano, Inc., Santa Barbara, CA, USA) under a tapping mode. The wet state of the membranes involved in the test is under a fully hydrated condition. The water uptake (WU), swelling ratio (SR), ion exchange capacities (IEC), proton conductivity (σ), methanol permeability (P), and selectivity (S) of the membranes were determined as reported elsewhere [14,17,18,21,22].

## 3. Results and Discussion

### 3.1. Characterization of NSGO

**Figure 1:**
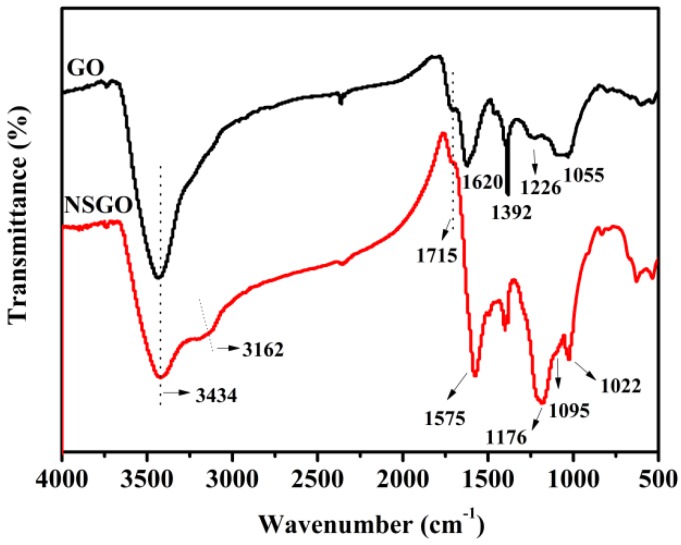
The FTIR spectra of GO and NSGO.

The FTIR spectra of GO and NSGO are displayed in Figure 1. The characteristic absorption peaks of GO appear at 1715, 1620, 1392, 1226, and 1055 cm^−1^, which correspond to the bands of C=O, C=C, C–O, epoxy C–O–C, and alkoxy C–O, respectively [23]. In addition, the characteristic absorption peak of –OH appears at 3434 cm^−1^ in the NSGO and GO spectra. Furthermore, the new peak at 3200 cm^−1^, which is present in the NSGO spectra and is not present in the GO spectra, is assigned to stretching vibrations of amino groups. Meanwhile, a strong absorption band that appeared at 1575 cm^−1^ corresponds to bending vibrations of amino groups [24]. Moreover, the characteristic absorption peak at 1176 cm^−1^ is ascribed to the C–N stretching vibration [24]. In addition, another two new peaks at 1095 and 1022 cm^–1^ are assigned to the asymmetric and symmetric stretching vibrations of sulfonic groups [18]. These prove that amino and sulfonic groups are successfully introduced into GO.

**Figure 2:**
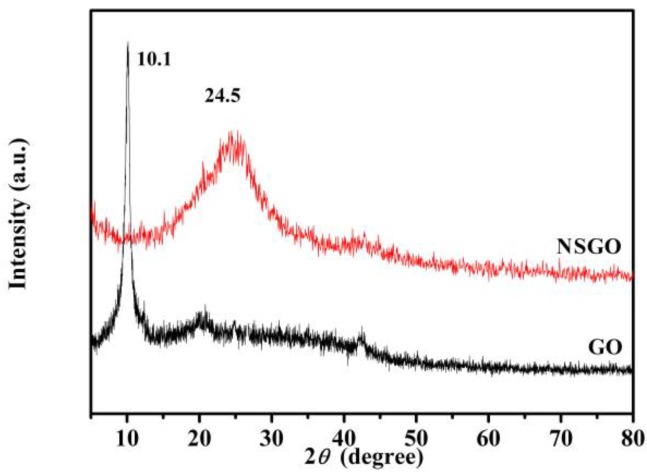
The XRD curves of GO and NSGO.

Figure 2 represents the XRD spectra of GO and NSGO. For GO, the sharp diffraction peak is observed at 10.1° (d = 0.87 nm). Moreover, the interlayer spacing of GO is much larger than that of graphite (2θ = 26.5°, d = 0.34 nm), owing to the introduction of oxygen containing functional groups (–COOH, –OH, and –CH(O)CH–) [25]. After reaction with aryl diazonium salt, the characteristic diffraction peak of GO disappears and a new diffraction peak at 2θ = 24.5° (d = 0.36 nm) appears. The functionalized GO layers with lower distance is mainly because it was further exfoliated and partially restacked through the π–π interaction and the removal of oxygen functional groups. This is consistent with the reported literature [25,26].

**Figure 3:**
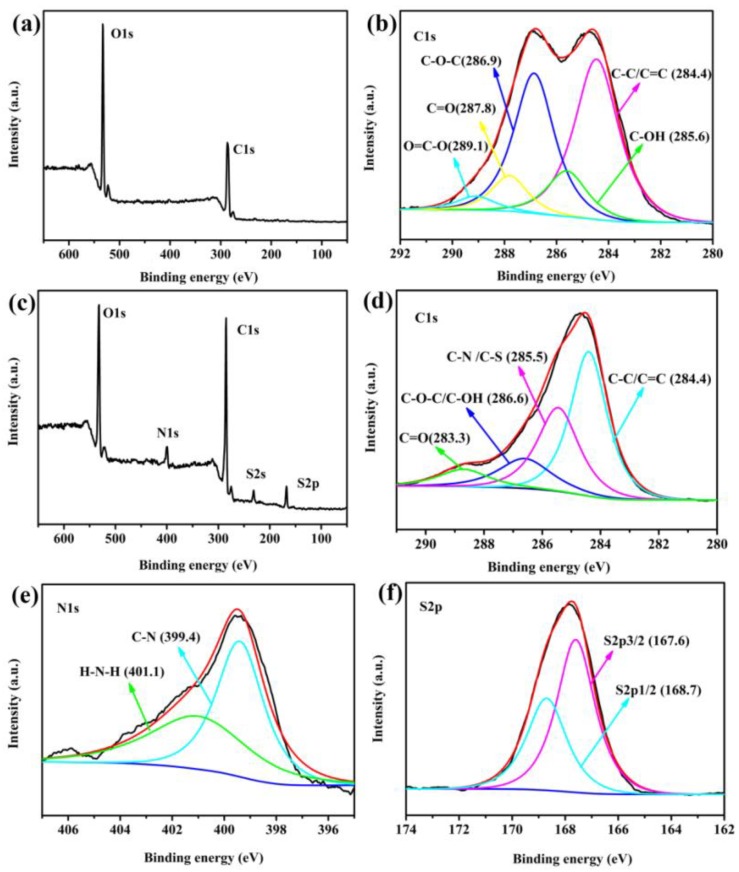
The XPS spectra of GO (**a**,**b**) and NSGO (**c**–**f**).

To further confirm the structure of GO and NSGO, the XPS spectra is recorded. As shown in Figure 3b, the C1s spectrum of GO shows five pronounced deconvoluted peaks at 284.4, 285.6, 286.9, 287.8, and 289.1 eV, corresponding to the C–C/C=C, C–OH, C–O–C, C=O, and O–C=O bonds, respectively [27]. By comparison, NSGO gives rise to new peaks at 399.8, 232.4, and 168.5 eV (Figure 3c), which is assigned to N1s, S2s, and S2p, respectively. The high-resolution XPS spectra of the C1s, N1s, and S2p regions of NSGO are showed in Figure 3d–f . It is clear that a new peak appears at 285.5 eV in the C1s region of NSGO, belonging to C–N and C–S bonds [28,29]. Moreover, the N1s region of NSGO exhibits two peaks at 399.4 eV for C–N bonds and at 401.1 eV for H–N–H bonds [24]. In addition, in the S2p spectrum of NSGO, two new peaks of S2p are observed at 167.6 and 168.7 eV, which is the typical representative of –SO_3_H groups [30]. These confirm the synthesis of functionalized GO containing both amino and sulfonic groups.

### 3.2. Characterization of the Membranes 

**Figure 4:**
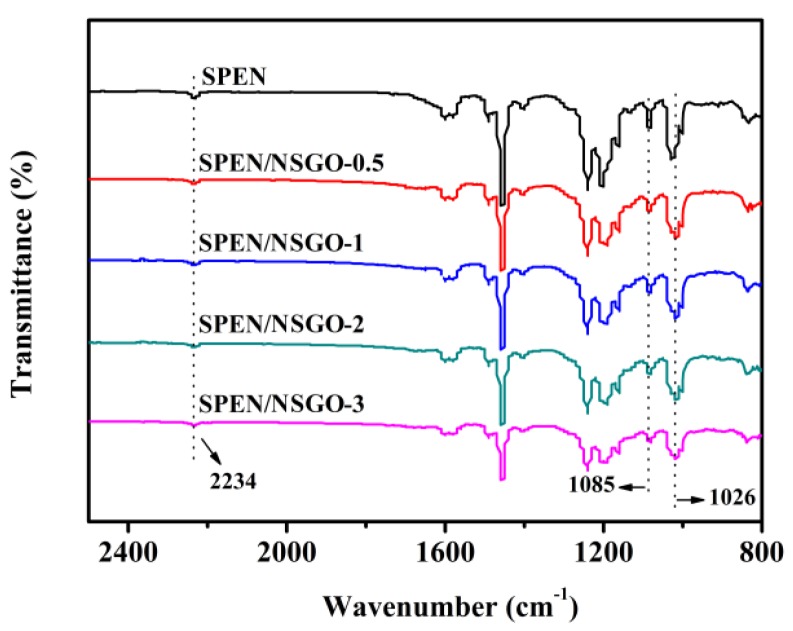
The FTIR spectra of the membranes.

The FTIR spectra of the SPEN and SPEN/NSGO composite membranes are shown in Figure 4. The characteristic absorption peak of cyano groups appears at 2234 cm^−1^ [31]. Additionally, the two absorptions at 1085 and 1026 cm^−1^ refer to the symmetric and asymmetric stretching vibrations of –SO_3_H. More particularly, the intensity of the sulfonic groups obviously decreases with the incorporation of NSGO (especially when the content of NSGO reaches 3 wt %), which is owing to the presence of acid–base interactions between the –SO_3_H groups and –NH_2_ groups [32].

### 3.3. SEM Images of the Membranes

**Figure 5:**
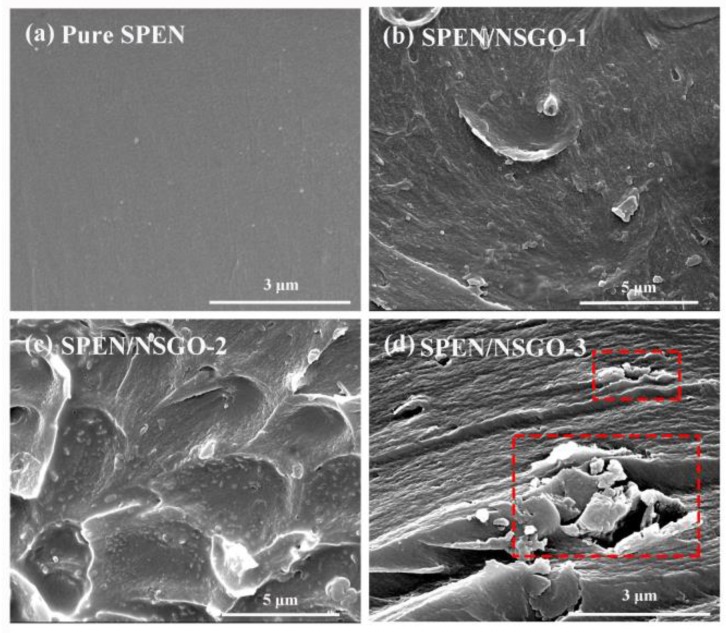
The SEM images of cross-sections of (**a**) SPEN, (**b**) NSGO/SPEN-1, (**c**) NSGO/SPEN-2, and (**d**) NSGO/SPEN-3 membranes. The red dashed line boxes indicate the defects of the membrane.

The cross-sectional morphologies of the SPEN and SPEN/NSGO composite membranes are probed by SEM. Figure 5a shows that the cross-sectional morphology of the SPEN membrane is smooth, dense, and macroscopically homogeneous. Compared to the SPEN membrane, the SEM images of the SPEN/NSGO composite membranes (Figure 5b,c) exhibit distinct differences. The cross-sectional morphologies of the SPEN/NSGO composite membranes become relatively rough due to the existence of NSGO, which is similar to findings in the previous literature [16,18]. Besides, it can be seen that embedded NSGO disperses homogeneously throughout the composite membranes, which is mainly attributed to its good compatibility with the SPEN matrix and the excellent interfacial interactions between the NSGO and SPEN matrix. Such excellent interactions are mainly derived from (1) the hydrogen bonding interactions between the –SO_3_H of NSGO and –SO_3_H of SPEN in the membranes, and (2) the acid–base interactions between the –NH_2_ of NSGO and –SO_3_H of the SPEN matrix [18]. Furthermore, when the content of NSGO increases to 3%, the cross-sectional morphology of the SPEN/NSGO composite membrane shows apparent defects, such as some cracks and pinholes, which result from the local aggregation of NSGO.

### 3.4. Thermal and Mechanical Stabilities of the Membranes

**Figure 6:**
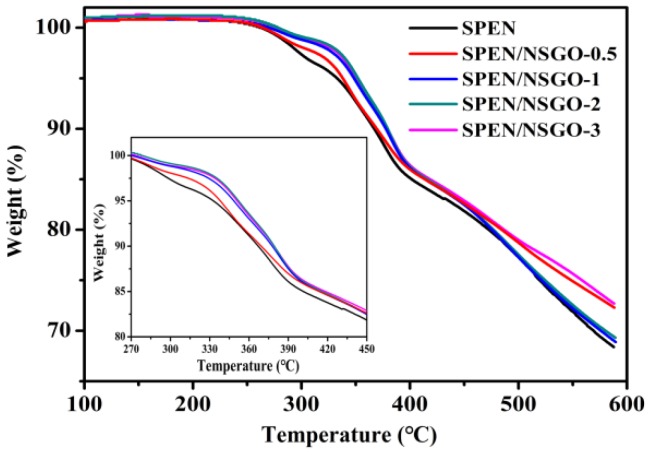
The TGA curves of the membranes under N_2_ atmosphere.

To ensure the long-time operation of proton exchange membrane, excellent thermal and mechanical stabilities are two indispensable factors. Figure 6 shows the TGA curves of all the membranes. These curves exhibit a similar degradation process, including two decomposition stages: one is attributed to the degradation of sulfonic groups around 270–330 °C; another stage over 400 °C is assigned to the decomposition of carbon main chains [22]. Moreover, the thermal stability of the SPEN/NSGO composite membranes can be enhanced by the introduction of NSGO, which is due to the strong interactions consisting of hydrogen bonding and acid–base interactions between the incorporated NSGO and SPEN matrix. Furthermore, the suppressive effect of the addition of NSGO on the diffusion of degradation products may be also beneficial to improve the thermal stability [33].

Mechanical stability of the membranes is represented in Table 1. It can be clearly seen that the SPEN membrane exhibits good mechanical properties, with a tensile strength and Young’s modulus of 52.96 and 766.03 Mpa, respectively. Furthermore, after introduction of NSGO, the tensile strength of the SPEN/NSGO composite membranes is increased to 57.98–70.55 Mpa, along with high Young’s modulus (825.06–896.04 Mpa). The improved mechanical properties are benefited by the excellent interfacial interactions (hydrogen bonding and acid–base interactions) between NSGO and SPEN. Besides, it is obviously observed that as NSGO content increases from 0.5 to 3 wt %, both the tensile strength and Young’s modulus first increase and then decrease. This reduction is mainly due to the aggregation of NSGO. Collectively, these data indicate that the SPEN/NSGO composite membranes have excellent thermal and mechanical stabilities for PEM applications.

### 3.5. Ion Exchange Capacities (IEC), Water Uptake (WU), and Swelling Ratio (SR) of the Membranes

IEC, reflecting the density of H^+^ exchangeable functional groups in the membrane, affects proton transport via the Grotthuss mechanism [18]. The IEC values of as-prepared membranes are obtained by the classical titration method and displayed in Table 2. The IEC values of the SPEN/NSGO composite membranes vary from 1.72–1.83 mmol·g^−1^, and show a slight increase compared to that of the SPEN membrane. The reason is that the additional –SO_3_H groups provided by NSGO increase the density of H^+^.

Usually, water molecules play an important role in PEMs, and thus can facilitate the proton transfer process through the formation of hydrogen bond networks [34]. However, too much water uptake will lead to excess swelling, making the membrane useless [22]. Table 2 presents the (a) WU and (b) SR of SPEN and the SPEN/NSGO composite membranes with different temperature in the fully hydrated condition. The WU and SR of all the membranes show a similar tendency with the increase of temperature. The SPEN membrane presents the WU of 58.02% and SR 16.72% at 80 °C. By comparison, the composite membranes show reduced WU (46.44%–56.12%) and SR (13.23%–16.24%). This may result from the strong mutual interactions between NSGO and the SPEN matrix that suppress the absorption of water. Although the extra sulfonic group of NSGO is provided, this is not enough to cause an increase in water absorption, suggesting that the acid–base interactions play a major role. The acid–base interactions effectively inhibit the movement of the chain, making the membrane more compact and leading to lower WU and SR. Therefore, with the increase of NSGO content, WU and SR decrease. Compared to that of SPEN/NSGO-2, SPEN/NSGO-3 shows a sharp increase in WU and SR, likely due to excessive filler content that causes the poor dispersion and compatibility with the matrix. The result is consistent with the cross-sectional morphologies of the SEM images. The aforesaid data suggest that the SPEN/NSGO composite membranes possess excellent dimensional stability, which is beneficial to the long-time use for PEMs.

### 3.6. Proton Conductivity of the Membranes

**Figure 7:**
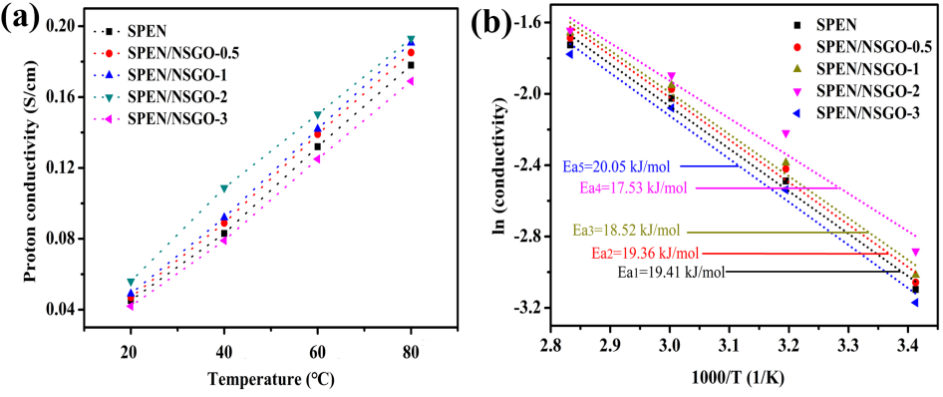
The (**a**) proton conductivity and (**b**) Arrhenius plots of the proton conduction of the membranes with different temperatures at 100% relative humidity (RH).

Proton conduction is a critical parameter for PEMs and directly relates to the performance of DMFCs. The proton conductivities of all the membranes with altered temperatures at 100% relative humidity (RH) are presented in Figure 7a. Obviously, proton conductivity is positively correlated with temperature because of the accelerated motion of water molecules and SPEN chains, implying that the proton transfer at the fully hydrated state is a thermally activated process [35]. Moreover, it is worth mentioning that the proton conductivity of all the SPEN/NSGO composite membranes is superior to that of the SPEN membrane. The high proton conductivity of the composite membranes is probably due to the acid–base pairs and extra sulfonic groups. For the SPEN/NSGO composite membranes, the incorporated NSGO can improve the proton conduction of the membrane until the content of NSGO reaches 2 wt %. However, when the NSGO loading is higher than 2 wt %, the aggregation effect of NSGO emerges, so the proton transfer channel in the composite membrane is blocked and thus the corresponding proton conductivity reduces [32]. This is in accordance with the SEM observation. Among all of the membranes, the SPEN/NSGO-2 membrane exhibits the highest proton conductivity and reaches up to 0.056 S·cm^−1^ at 20 °C and 0.193 S·cm^−1^ at 80 °C. Considering the fact that NSGO reduces the WU and SR of the composite membranes, the increase of proton conductivity is mainly related to the increase of the Grotthuss-type mechanism in proton transport [36].

For further studying the proton transfer mechanism, the values of activation energy (E_a_) for proton conduction can be calculated using the Arrhenius equation, as depicted in Figure 7b. The E_a_ values of all the membranes are in the range of 17.53–20.05 kJ·mol^−1^. As is known, the E_a_ value of the Grotthuss mechanism is in the range of 14.3–39.8 kJ·mol^−1^ [37]. Our result suggests that the Grotthuss mechanism is the main path of proton transfer in the membranes. Moreover, NSGO provides additional proton hopping sites (sulfonic groups and acid–base pairs) for the composite membranes, resulting in the reduction of the E_a_ value. This will allow more protons to migrate via the Grotthuss mechanism, which is in agreement with the proton conductivity analysis and previous works [15,18].

**Figure 8:**
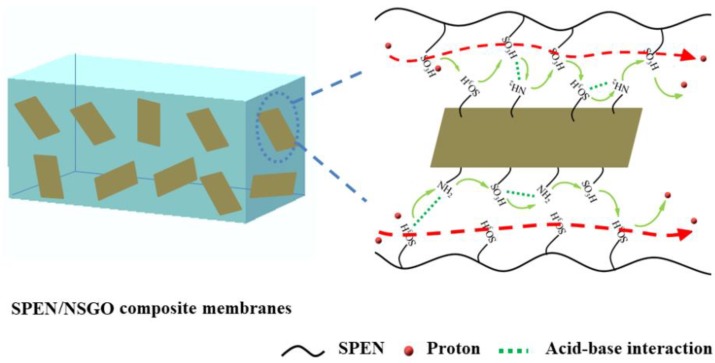
The possible proton transfer mechanism of the composite membranes.

The detailed mechanism of proton conduction in the SPEN/NSGO composite membranes is proposed in Figure 8. Generally, proton conduction is mainly dependent on the vehicle mechanism and the Grotthuss mechanism [38,39]. For the composite membranes, according to the above discussion, we can know that the Grotthuss mechanism is involved as the main mechanism; that is to say, H^+^ jumps to adjacent groups through the protonation/deprotonation process [40]. SPEN containing abundant sulfonic groups can easily form local ion clusters, and then facilitate the proton transfer via the Grotthuss mechanism. After NSGO is introduced into the SPEN matrix, the microstructure of the membranes is rearranged by NSGO, creating more new proton-conducting paths. Owing to the abundant sulfonic and amino groups of NSGO, hydrogen-bonding interactions and acid–base interactions with the ionic clusters of the SPEN matrix are formed, providing more proton-hopping sites and connecting ion clusters. On the one hand, the protons can be transferred continuously along the SPEN/NSGO interface via the Grotthuss mechanism. This may make the proton transport channel more continuous, promoting the proton conductivity. On the other hand, some protons also may transfer on the edge and surface of NSGO via the Grotthuss mechanism. This is mainly due to NSGO containing sulfonic and amino groups, which can effectively shorten the distance of proton transfer. It also contributes to the formation of continuous proton channels. Furthermore, the reduced E_a_ confirms that effective proton transfer occurs mainly through a low-energy-barrier manner. Thus, high-speed channels are built for proton conducting. In short, the introduction of NSGO provides more additional proton hopping sites and constructs continuous proton-conducting highways along the SPEN/NSGO interface, ensuring the improvement of the proton conductivity.

**Figure 9:**
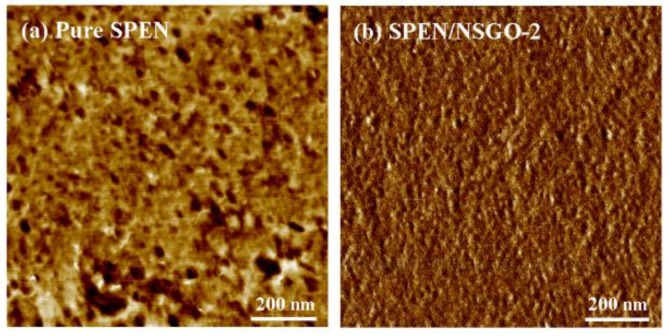
The AFM phase images of (**a**) SPEN and (**b**) SPEN/NSGO-2 composite membranes.

To further study the reasons for the increase of proton conductivity, the microscopic morphologies of the membranes are examined by AFM. Figure 9 shows the tapping-mode images of these membranes. The dark regions in the phase images represent the soft parts of hydrophilic domains, and bright regions are related to the hard block of hydrophobic domains [41]. Thus, the dark region can be regarded as the formation of ion channels. The ionic clusters of the SPEN membrane are relatively disconnected, while the SPEN/NSGO-2 membrane shows narrow and continuous ion channels. This further demonstrates that the addition of NSGO is beneficial to proton transfer, which is in good agreement with the proton conductivity results and confirms the abovementioned speculation.

### 3.7. Methanol Permeability and Selectivity of the Membranes

Methanol permeability is another critical factor to evaluate the performance of DMFCs. Since protons and methanol share the same channels, there is a trade-off effect between proton conduction and methanol permeability [42]. The ideal PEMs should have high proton conductivity and low methanol permeation, simultaneously. As shown in Table 3, the methanol permeation levels of the composite membranes are all lower than that of the SPEN membrane. It is due to the fact that the large surface area and barrier effect of NSGO can increase the tortuosity of transport channels, resulting in a longer path and a decrease in methanol permeability [17,43]. Meanwhile, the strong acid–base interactions between NSGO and the SPEN matrix are favorable for forming a more compact structure, and also inhibiting methanol permeability. The SPEN/NSGO-2 membrane exhibits the lowest methanol permeability (1.41 × 10^−7^ cm^2^·s^−1^), which is far lower than that of the SPEN membrane (4.12 × 10^−7^ cm^2^·s^−1^).

Selectivity (the ratio of proton conductivity and methanol permeability) is used to forecast the performance of DMFCs. Generally, the membrane with higher selectivity also has better battery performance [32,40]. Table 3 lists the selectivity of the SPEN and SPEN/NSGO composite membranes. The selectivity of all the composite membranes is higher than that of the SPEN membrane. In particular, the selectivity of the SPEN/NSGO-2 membrane reaches up to 3.97 × 10^5^ S·cm^−3^·s, which is 3.6 times higher than that of the SPEN membrane (1.09 × 10^5^ S·cm^−3^·s). Moreover, the selectivity of the composite membranes is better than in other reported works [14,18,19,21,44] (see Table 3). Furthermore, the optimal selectivity of amino-sulfo-bifunctionalized GO (NSGO) as the filler is better than that of sulfonic-functionalized GO or amino-functionalized GO. These demonstrate that our work describes an accessible and effective method to prepare high-performance PEMs.

## 4. Conclusions

In this work, amino-sulfo-bifunctionalized GO was prepared through the reaction of aryl diazonium salt and then incorporated into a SPEN matrix to fabricate composite membranes via the solution-casting method. The as-obtained composite membranes display excellent dimensional stability and thermal and mechanical properties induced by good interfacial interactions, consisting of acid–base and hydrogen-bonding interactions between NSGO and the SPEN matrix. In addition, continuous proton-conducting highways are constructed along the SPEN/NSGO interface, which are beneficial to enhance proton transfer. Moreover, the composite membranes are endowed with low methanol permeability due to the barrier effect of NSGO and acid–base interactions. As a consequence, the SPEN/NSGO-2 composite membrane shows the highest selectivity of 3.97 × 10^5^ S·cm^−3^·s, which is 3.6 times higher than that of the SPEN membrane. The present study provides a new strategy for constructing continuous proton-conduction channels within a composite membrane to obtain high-performance PEMs.

## Figures and Tables

**Table 1:** Mechanical stability of the membranes tested in the fully hydrated condition at room temperature.

Membranes	Tensile strength (MPa)	Tensile modulus (MPa)	Elongation at break (%)
SPEN	52.96 ± 1.96	766.03 ± 20.13	84.87 ± 3.01
SPEN/NSGO-0.5	57.98 ± 1.78	825.06 ± 20.21	75.42 ± 3.32
SPEN/NSGO-1	63.82 ± 1.53	872.87 ± 24.79	71.10 ± 2.72
SPEN/NSGO-2	70.55 ± 2.90	896.04 ± 25.63	67.03 ± 2.24
SPEN/NSGO-3	64.26 ± 2.49	881.83 ± 23.04	62.05 ± 2.57

**Table 2:** Ion exchange capacities (IEC), water uptake (WU), and swelling ratio (SR) of the membranes.

Membranes	IEC (mmol/g)	WU (%)				SR (%)			
		20 °C	40 °C	60 °C	80 °C	20 °C	40 °C	60 °C	80 °C
SPEN	1.76 ± 0.01	20.05	27.05	37.39	58.02	7.02	8.12	11.37	16.72
SPEN/NSGO-0.5	1.77 ± 0.01	19.89	26.29	36.95	56.12	6.79	7.54	10.79	16.24
SPEN/NSGO-1	1.80 ± 0.02	17.24	24.82	35.08	50.09	6.34	7.36	9.91	14.75
SPEN/NSGO-2	1.83 ± 0.02	15.05	23.17	32.72	46.44	5.11	6.52	8.79	13.23
SPEN/NSGO-3	1.72 ± 0.01	17.43	25.11	34.05	47.48	6.13	7.29	10.41	14.98

**Table 3:** Transport properties of the membranes at room temperature.

Membranes	Proton conductivity (S·cm^−1^)	Methanol permeation (10^−7^ cm^2^·s^−1^)	Selectivity (10^5^ S·cm^−3^·s)
SPEN	0.045	4.12	1.09
SPEN/NSGO-0.5	0.047	2.21	2.13
SPEN/NSGO-1	0.049	1.61	3.04
SPEN/NSGO-2	0.056	1.41	3.97
SPEN/NSGO-3	0.042	1.87	2.25
SPEN/SGO-2 [14]	0.0589	3.126	1.884
S [18]	0.0505	2.49	2.04
N [18]	0.053	1.25	2.46
SPEEK/SDBS-GO (8 wt %) [19]	0.0938	9.5	0.99
SPEN/SCNTs/SPEN (3 wt %) [21]	0.094	8.0	1.175
Nafion 117 [21]	0.064	14.1	0.45
SGO-5 [44]	0.058	1.556	3.727
